# Comparison of the Effectiveness of Online and Face-to-Face Parent Training for Parents of Children With Developmental Disabilities

**DOI:** 10.7759/cureus.73895

**Published:** 2024-11-18

**Authors:** Ryuki Kadekaru, Tomohisa Yamanaka, Tohru Okanishi, Yoshihiro Maegaki, Masahiko Inoue

**Affiliations:** 1 Department of Doctoral Course, Graduate School of Medical Sciences, Tottori University, Yonago, JPN; 2 Advanced Medicine, Innovation and Clinical Research Center, Tottori University Hospital, Yonago, JPN; 3 Student Accessibility Office, Head Office for Education and Student Support, Shimane University, Matsue, JPN; 4 Department of Brain and Neurosciences, Division of Child Neurology, Faculty of Medicine, Tottori University, Yonago, JPN; 5 Department of Clinical Psychology, Graduate School of Medical Sciences, Tottori University, Yonago, JPN

**Keywords:** behavioral interventions, behavioral problem, developmental disabilities, internet-based intervention, parent training

## Abstract

Background: Parent training (PT) is an effective intervention for improving children's behavioral problems and enhancing parental mental health in those caring for children with developmental disabilities (DD). Recent studies report the effectiveness of online PT (ON-PT). ON-PT encompasses both the on-demand type and the real-time type, which involves real-time online group PT delivered through web conferencing systems. However, the efficacy of the on-demand type has been established through comparisons with face-to-face PT (F2F-PT), whereas the real-time type of ON-PT has been assessed exclusively in single-arm studies, underscoring the need for comparative analyses with F2F-PT to validate its effectiveness. This study aims to compare the effectiveness of the real-time type of ON-PT and F2F-PT for parents of children with DD using a retrospective study design.

Methods: The analysis included data from 13 parent-child pairs in the F2F-PT and 27 parent-child pairs in the ON-PT. Assessment scales included parental depression and stress, evaluated using the Beck Depression Inventory-Second Edition (BDI-II) and Parenting Stress Index (PSI), respectively, as well as children's behavioral problems, measured with the Eyberg Child Behavior Inventory (ECBI). A two-way repeated-measures ANOVA assessed the impact of different PT delivery methods and time on the outcome variables.

Results: Attendance and dropout rates were similar between ON-PT (82%, 18.7%) and F2F-PT (80.3%, 18.1%). A two-way repeated-measures ANOVA showed that the interaction effect was marginally significant for PSI (*p* = 0.066) and statistically significant for both the child domain of PSI (*p* = 0.049) and ECBI (*p* = 0.013). Simple main effects analysis indicated that pre-test mean scores for PSI (*p* < 0.001), the child domain of PSI (*p* = 0.001), and ECBI (*p* = 0.002) were significantly higher than post-test scores in the ON-PT compared with the F2F-PT. Furthermore, although a higher proportion of participants in the ON-PT were within the clinical range of ECBI at the pre-test (70.4%) compared to the F2F-PT, this proportion decreased to 44.4% at the post-test.

Conclusion: This study suggests that ON-PT may be as effective as or potentially more effective than F2F-PT. The adoption of online formats should be considered for families facing challenges, as ON-PT may improve children's behavioral problems and reduce parental stress. Nonetheless, the retrospective study design warrants caution in interpreting the findings, and a future study with a prospective, rigorous validation design will be essential to effectively compare the effectiveness of ON-PT and F2F-PT.

## Introduction

Children with developmental disabilities (DD) are at a heightened risk of behavioral problems [1], and the prevalence of these conditions is rising, with an increase from 12.84% to 15.04% over the past 12 years [2]. Meta-analyses have shown that parents of children aged 0-5 years with DD have a higher likelihood of mental health disorders, including depressive symptoms and stress (standardized mean difference (SMD) = 0.87; 95% predictive interval, -0.47 to 2.22), suggesting poorer overall health compared to parents of typically developing children [3]. The World Health Organization recommends prioritizing access to appropriate community support and services for children with DD and their parents [4].

Review studies, including 17 controlled trials, demonstrate that parent intervention programs significantly improve behavioral problems in children aged 3-8 years with DD, with effect sizes (ES) ranging from moderate to large [5]. Parent training (PT) is internationally recommended to enhance child adjustment and behavior, as well as to improve parenting skills and parental mental health [6]. PT is typically provided to parents of children with attention-deficit/hyperactivity disorder (ADHD), autism spectrum disorder (ASD), and intellectual disability (ID) based on disability type, with systematic reviews and meta-analyses demonstrating its effectiveness [7-9]. Rimestad et al. [7] reported moderate ES for PT in children with ADHD, with reductions in ADHD symptoms (ES = 0.51), conduct problems (ES = 0.40), and negative parenting behaviors (ES = 0.63). Postorino et al. [8] found a moderate effect of PT in reducing disruptive behaviors in children with ASD (SMD = -0.59; 95% confidence interval, -0.88 to -0.30; *p* < 0.001). For children with ID, Coren et al. [9] reported some improvements in parenting skills, though low-quality evidence limits generalizability.

The COVID-19 pandemic has increased expectations for online PT (ON-PT) as an adaptation of traditional face-to-face PT (F2F-PT) [10]. ON-PT is particularly valuable for families with limited access to traditional F2F-PT [11]. Therefore, ON-PT is an important method for delivering PT to a broader range of areas.

ON-PT studies have been validated through comparisons with F2F-PT [12]. However, the only ON-PT studies comparing to F2F-PT involve on-demand type, where web-based content is accessed and progressed voluntarily [12]. Although on-demand type is accessible at any time, it provides limited opportunities for interaction with other participants [13,14] and poses challenges in maintaining participant motivation [15].

ON-PT encompasses both the on-demand type and the real-time type, which involves real-time online group PT delivered through web conferencing systems [16-18]. The real-time type of ON-PT, similar to conventional F2F-PT, facilitates interaction between therapists and participants and incorporates group work exercises. Review studies comparing individual and group formats of PT indicate that bidirectional elements, such as parent-to-parent interactions in group formats like F2F-PT, are essential for enhancing treatment engagement and supporting favorable therapeutic outcomes [19]. Although the real-time type of ON-PT incorporates interactive elements similar to those in F2F-PT, its efficacy remains unconfirmed due to a lack of comparative studies [16-18]. Thus, a comparative study between ON-PT and F2F-PT is needed to establish the effectiveness of the real-time type of ON-PT.

Previously, we implemented F2F-PT and the real-time type of ON-PT program, each with identical content, to teach adaptive skills such as daily living and communication to parents of children aged 3-9 years with DD for use in the home setting. This study aims to compare improvements in parental mental health and children's behavioral problems between these two types of PT using a retrospective study design. This analysis provides preliminary insights into the effectiveness of F2F-PT and ON-PT, establishing a foundation for future prospective studies to validate and expand these findings.

## Materials and methods

Participants

This study utilized data from PT we conducted with Japanese parents of children with DD. These PTs were conducted over multiple years, with F2F-PT delivered in 2017 and 2018 and ON-PT implemented in 2020 and 2021 via a web conferencing system under COVID-19 restrictions. F2F-PT participants were recruited by distributing leaflets to parents of children aged 3-9 years with a diagnosis or suspected diagnosis of DD who attended hospitals and day services in regions offering developmental support. For ON-PT, parents of children aged 3-9 years with a diagnosis or suspected diagnosis of DD were recruited through leaflets distributed to healthcare providers and made available on a website. ON-PT also required participants to have access to a web conferencing platform (Zoom) as part of the eligibility criteria.

PT practitioners

The PT practitioners included a graduate student specializing in clinical psychology, a research student focusing on special education, and an experienced parenting mentor for DD. Graduate and research students served as "main facilitators" for lectures and "group facilitators" for group activities. The parenting mentor was responsible for sharing their experiences with DD parenting. ON-PT appointed a graduate student as a "network manager" to maintain internet stability and provide technical support. The overall process was supervised by the last author, who provided guidance and oversight to the PT practitioners.

PT program

The PT program was structured based on the Tottori University parent training (TUPT) program [16,18,20], which has demonstrated efficacy in previous studies and was designed to help parents practice appropriate interactions with their children and teach adaptive skills (such as daily living skills and communication) at home, using applied behavior analysis techniques. The F2F-PT sessions were held in a room at Tottori University, while the ON-PT sessions were conducted via Zoom and consisted of eight 120-minute group sessions held biweekly. Table 1 provides an overview of the PT program.

**Table 1:** Overview of the parent training program.

Session	Lecture	Group work	Homework assignment
1	Orientation. How to praise effectively. Relaxation	Sharing daily praise. Considering the praise words that children will enjoy	Practice praise for children. Record the circumstances when the children are praised, the method of praise used, and the children's response
2	Reframing. Functional analysis. Relaxation	Sharing homework. Reframing	Practice praising children in a way that makes them happy. Record how children respond to praise
3	Token economy. Functional analysis. Relaxation	Sharing homework. Applying praise in a hypothetical scenario. Performing functional analysis in a hypothetical scenario	Perform and record the instructional task
4	Environmental modifications to encourage adaptive behavior. Relaxation	Sharing homework. Practicing environmental adaptation through a hypothetical case	Consider effective environmental modifications for instructional tasks
5	Communication strategies to promote adaptive behavior. Relaxation	Sharing homework. Setting up the task sheet	Engage in communication practices to encourage adaptive behavior. Record examples of communication methods practiced and children's reactions to them
6	Task analysis. Prompting. Relaxation	Sharing homework. Setting up the task sheet	Conduct a structured instructional task. Record the outcomes of the practice session
7	Prompting. Relaxation	Sharing homework. Setting up the task sheet	Conduct a structured instructional task. Record the outcomes of the practice session
8	Graduation ceremony. How to promise children things. Relaxation	Sharing homework	

The lecture sessions covered five topics: effectively praising children's behavior, conducting a functional analysis of inappropriate behavior, adjusting the environment to minimize inappropriate behavior, communicating with children displaying inappropriate behavior, and performing task analysis and prompting when teaching adaptive behavior.

Group work was conducted in small groups of 3-4 participants alongside PT practitioners. Each group discussed session-related issues and themes 2-3 times per session, with each discussion lasting 10-20 minutes. Afterward, the staff members shared the small groups' opinions and questions with the entire group. ON-PT utilized Zoom's "Breakout Room" feature to replicate the group work experience of F2F-PT. Participants were also encouraged to ask questions about the PT program content as needed.

Participants in the study were required to complete at least six of the eight sessions in the PT program. The program was delivered in two formats over different periods: F2F-PT sessions pre-COVID-19 (2017-2018) and ON-PT sessions during the COVID-19 era (2020-2021). Session 1 focused on orientation and self-introductions, followed by teaching participants on how to effectively praise their children. Session 2 involved sharing and discussing homework related to praising children. In session 3, participants practiced praise techniques through role-playing with fictional cases. Group work included reporting on homework outcomes and practicing functional assessment using fictional cases. Session 4 introduced materials for environmental adjustments, while session 5 discussed strategies for adjusting the environment and proper levels of instruction. Sessions 6 and 7 focused on setting up and implementing instructional tasks with the support of PT practitioners. In session 8, participants presented their homework results, followed by a lecture from the PT practitioners on making promises to children. The program concluded with a graduation ceremony during which participants reflected on what they had learned and practiced. To help participants relax and fully engage in the PT program, relaxation techniques were introduced at the beginning of each session, utilizing breathing exercises in F2F-PT and mindfulness practices in ON-PT. Both PT programs were largely similar; however, the ON-PT program required the use of specific Zoom features, such as screen and audio switching, and included different relaxation techniques.

Assessment scales

Parental Mental Health

The Beck Depression Inventory-Second Edition (BDI-II) is a standardized assessment instrument designed to assess depressive symptoms through 21 items that evaluate the severity of depression [21]. This study used the Japanese version of the BDI-II [22], which has been validated for reliability and validity. In accordance with the Japanese version guidelines, a 0-3-point Likert scale was used, with total scores ranging from 0 to 63. The cut-off scores were defined as follows: 0-13, for minimal depression; 14-19, for mild depression; 20-28, for moderate depression; and 29-63, for severe depression. The total scores were used for analysis in this study. Parents assessed their depressive symptoms at the beginning of the first session and after the completion of the eighth session.

The Parenting Stress Index (PSI) is a standardized assessment instrument designed to assess parenting stress through 78 items, covering two primary domains: the parent domain (40 items across eight subscales) and the child domain (38 items across seven subscales) [23]. This study used the Japanese version of the PSI [24], which has been validated for reliability and validity. A 1-5-point Likert scale was used in accordance with the Japanese version guidelines. The cut-off scores were defined as follows: a total score of 221 or higher, a parent domain score of 124 or higher, and a child domain score of 101 or higher. The score ranges were set as 78-390 for the total score, 40-200 for the parent domain, and 38-190 for the child domain. The total scores were used for analysis in this study. Parents assessed their stress at the beginning of the first session and after the completion of the eighth session.

Children's Behavioral Problems

The Eyberg Child Behavior Inventory (ECBI) is a standardized assessment instrument designed to assess children's inappropriate daily behavior and caregivers' difficulties in raising their children through 36 items [25]. This study used the Japanese version of the ECBI [26], which has been validated for reliability and validity. In accordance with the Japanese version guidelines, a 1-7-point Likert scale was used, with total scores ranging from 0 to 252. The cut-off score was set at 125 points, indicating that children scoring 125 or higher were considered to have behavioral problems within the clinical range [26,27]. Parents assessed their children's behavior at the beginning of the first session and after the completion of the eighth session.

Data analyses

To analyze the participants' profiles, independent *t*-tests were conducted for age and homework submission frequency, while Fisher's exact test was used for other variables. Inclusion criteria for the analysis included participants aged 20 years or older who had children with DD aged 3-9 years who participated in the PT program. Exclusion criteria for analysis included participants who did not complete the pre-intervention (pre-test) and post-intervention (post-test) questionnaires or who attended fewer than six of the eight sessions (dropouts). A two-way repeated measures ANOVA (RM-ANOVA) was conducted to investigate changes in parental mental health and children's behavioral problems by assessing the impact of PT delivery conditions (F2F-PT and ON-PT) and time (pre- and post-tests) on the outcome variables. Cut-off scores were calculated for each measure to determine the number and percentage of participants in each group meeting clinical criteria. A *p*-value of less than 0.05 was considered statistically significant, while a *p*-value of less than 0.10 was considered marginally significant. All statistical analyses were conducted using R software, version 4.3.3 (R Foundation for Statistical Computing, Vienna, Austria).

## Results

Demographic data of the participants

Of the 51 participant pairs (one parent and one child each), two pairs from ON-PT who did not complete the pre- and post-test questionnaires and nine pairs (three from F2F-PT and six from ON-PT) who attended fewer than six of the eight sessions were excluded from the analysis. Before excluding these participants, the attendance rates were 82% for F2F-PT and 80.3% for ON-PT, while the dropout rates were 18.7% for F2F-PT and 18.1% for ON-PT.

Data from 40 participant pairs (13 pairs from F2F-PT and 27 pairs from ON-PT) were used for analysis. The participants' demographic data are presented in Table 2. There were no significant group differences in the parents' age (*t* = 0.195; *p* = 0.847) or gender (*p* = 0.307) nor in the number of homework submissions (*t* = 0.203; *p* = 0.841). For the children, there were no significant group differences in age (*t* = 1.390; *p* = 0.173), age groups (*p* = 0.090), gender (*p* = 0.226), or diagnosis (*p* = 0.089).

**Table 2:** Demographic data of the participants (n = 40). F2F-PT: face-to-face parent training; ON-PT: online parent training; ADHD: attention-deficit/hyperactivity disorder; ASD: autism spectrum disorder; DD: developmental disability

Variable	F2F-PT (*n* = 13)	ON-PT (*n* = 27)	t	p
Parents				
Age (years)			0.195	0.847
M	40.08	40.37		
SD	4.38	4.51		
Gender (*n*)				0.307
Male	3	2		
Female	10	25		
Homework submissions			0.203	0.841
M	5.08	5.22		
SD	1.98	2.19		
Children				
Age (years)			1.390	0.173
M	6.54	5.58		
SD	1.27	1.76		
Gender (*n*)				0.226
Male	12	19		
Female	1	8		
Age groups (*n*)				0.090
Preschool age	2	12		
School age	11	15		
Diagnosis (*n*)				0.089
ASD	4	15		
ADHD	2	2		
ASD, ADHD	3	1		
Down syndrome	1	0		
Suspected DD	3	9		

Parental mental health

Total Score of BDI-II

The results of the analysis for the total scores are presented in Table 3. The two-way RM-ANOVA revealed a significant main effect of group and time, but the interaction effect was not significant (group: *F* (1, 38) = 4.142; *p* = 0.048; *η²* = 0.098; time: *F* (1, 38) = 9.470; *p* = 0.003; *η²* = 0.199; interaction: *F* (1, 38) = 1.127; *p* = 0.295; *η²* = 0.028).

**Table 3:** Effects on BDI-II, PSI, and ECBI outcome measures by pre- and post-tests. ^***^p < 0.001;^ **^p < 0.01; ^*^p < 0.05; ^†^*p* < 0.10; effect sizes were categorized as follows: small: 0.01 ≤ *η*^2^ < 0.06; medium: 0.06 ≤ *η*^2^ < 0.14; large: 0.14 ≤ *η*^2^. F2F-PT: face-to-face parent training; ON-PT: online parent training; BDI-II: Beck Depression Inventory-Second Edition; ECBI: Eyberg Child Behavior Inventory; PSI: Parenting Stress Index

			F2F-PT (*n* = 13)		ON-PT (*n* = 27)		Main effect group			Main effect time			Group × time interaction		
			Pre-test	Post-test	Pre-test	Post-test	F	p	η^2^	F	p	η^2^	F	p	η^2^
BDI-II	Total score	M	10.69	8.08	18.74	13.37	4.142	0.048^*^	0.098	9.470	0.003^**^	0.199	1.127	0.295	0.028
		SD	(7.46)	(6.55)	(11.94)	(11.42)									
PSI	Total score	M	199.38	196.46	249.92	231.59	13.789	0.000^***^	0.266	6.774	0.013^*^	0.151	3.560	0.066^†^	0.085
		SD	(26.75)	(38.85)	(37.83)	(37.17)									
	Parent domain	M	101.76	98.92	131.11	121.33	10.568	0.002^**^	0.217	6.213	0.017^*^	0.140	1.873	0.179	0.047
		SD	(20.31)	(25.27)	(24.99)	(26.06)									
	Child domain	M	97.61	97.69	118.81	110.25	12.425	0.001^**^	0.246	3.966	0.053^†^	0.094	4.111	0.049^*^	0.097
		SD	(10.72)	(15.90)	(16.28)	(16.41)									
ECBI	Total score	M	111.15	114.23	134.00	119.70	3.631	0.064^†^	0.087	2.796	0.102	0.068	6.704	0.013^*^	0.150
		SD	(19.78)	(25.27)	(26.33)	(23.14)									

First, a main effect test of the group factor revealed a significant difference at the pre-test (*F* (1, 38) = 4.936; *p* = 0.032). Multiple comparisons using Bonferroni's method indicated that the mean scores for ON-PT were significantly higher than those for F2F-PT at the pre-test.

Second, a main effect test of the time factor (pre- and post-tests) at each level of the provision condition factor (F2F-PT and ON-PT) showed a marginal significance for F2F-PT (*F* (1, 12) = 3.616; *p* = 0.081) and a significant difference for ON-PT (*F* (1, 26) = 10.381; *p* = 0.003). Multiple comparisons using Bonferroni's method revealed that the mean scores were significantly higher at the pre-test than at the post-test.

Percentage of Cut-Off (BDI-II)

The proportions of participants meeting the cut-off scores are presented in Table 4. For "minimal", the number of participants in the F2F-PT increased from eight (61.53%) at the pre-test to 10 (76.92%) at the post-test, and in the ON-PT, the number increased from 11 (40.74%) at the pre-test to 15 (55.55%) at the post-test. For "mild depression", the number of participants in the F2F-PT decreased from four (30.76%) at the pre-test to two (15.38%) at the post-test, and in the ON-PT, the number increased from three (11.11%) at the pre-test to seven (25.92%) at the post-test. For "moderate depression", the number of participants in the F2F-PT increased from zero (0.00%) at the pre-test to one (7.69%) at the post-test, and in the ON-PT, the number decreased from eight (29.62%) at the pre-test to three (11.11%) at the post-test. For "severe depression", the number of participants in the F2F-PT decreased from one (7.69%) at the pre-test to zero (0.00%) at the post-test, and in the ON-PT, the number decreased from five (18.51%) at the pre-test to two (7.40%) at the post-test.

**Table 4:** Percentage of cut-offs on BDI-II, PSI, and ECBI outcome measures by pre- and post-tests. F2F-PT: face-to-face parent training; ON-PT: online parent training; BDI-II: Beck Depression Inventory-Second Edition; ECBI: Eyberg Child Behavior Inventory; PSI: Parenting Stress Index

		F2F-PT (*n *= 13)		ON-PT (*n* = 27)	
		Pre-test	Post-test	Pre-test	Post-test
BDI-II					
Minimal depression	*n* (%)	8 (61.53)	10 (76.92)	11 (40.74)	15 (55.55)
Mild depression	*n* (%)	4 (30.76)	2 (15.38)	3 (11.11)	7 (25.92)
Moderate depression	*n* (%)	0 (0.00)	1 (7.69)	8 (29.62)	3 (11.11)
Severe depression	*n* (%)	1 (7.69)	0 (0.00)	5 (18.51)	2 (7.40)
PSI					
Clinical cut-off	*n* (%)	4 (30.76)	4 (30.76)	22 (81.48)	19 (70.37)
PSI (parent domain)					
Clinical cut-off	*n* (%)	2 (15.38)	3 (23.07)	15 (55.55)	19 (70.37)
PSI (child domain)					
Clinical cut-off	*n* (%)	4 (30.76)	5 (38.46)	25 (92.59)	24 (88.88)
ECBI					
Clinical cut-off	*n* (%)	4 (30.76)	6 (46.15)	19 (70.37)	12 (44.44)

Total Score of PSI

The results of the analysis for the total scores are presented in Table 3. The two-way RM-ANOVA revealed a significant main effect of group and time, with a marginally significant interaction effect (group: *F* (1, 38) = 13.789; *p* < 0.001; *η²* = 0.266; time: *F* (1, 38) = 6.774; *p* = 0.013; *η²* = 0.151; interaction: *F* (1, 38) = 3.560; *p* = 0.066; *η²* = 0.085).

**Figure 1:**
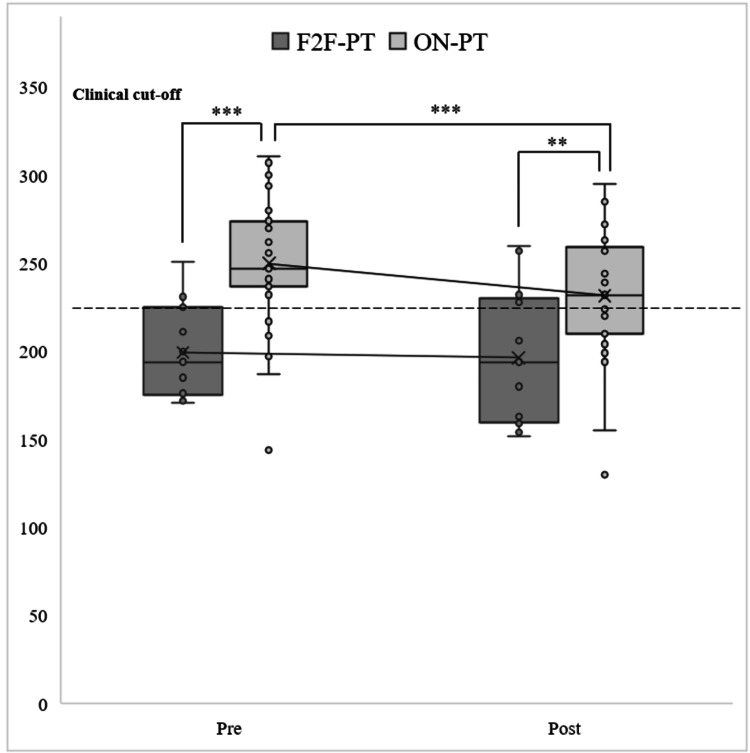
Multiple comparisons using Bonferroni's method (PSI). ^***^p < 0.001;^ **^p < 0.01. F2F-PT: face-to-face parent training; ON-PT: online parent training; PSI: Parenting Stress Index

First, a simple main effect test of the group factor revealed a significant difference at the pre-test (*F* (1, 38) = 18.594; *p* < 0.001) and post-test (*F* (1, 38) = 7.613; *p* = 0.008). Multiple comparisons using Bonferroni's method indicated that the mean scores for ON-PT were significantly higher than those for F2F-PT at the pre- and post-tests (Figure 1).

Second, a simple main effect test of the time factor (pre- and post-tests) at each level of the provision condition factor (F2F-PT and ON-PT) showed no significant difference for F2F-PT (*F* (1, 12) = 0.173; *p* = 0.684), but a significant difference for ON-PT (*F* (1, 26) = 16.209; *p* < 0.001). Multiple comparisons using Bonferroni's method revealed that the mean scores were significantly higher at the pre-test than at the post-test (Figure 1).

Parent Domain of PSI

The results of the analysis for the parent domain of PSI are presented in Table 3. The two-way RM-ANOVA revealed significant main effects of group and time, but the interaction effect was not significant (group: *F* (1, 38) = 10.568; *p* = 0.002; *η²* = 0.217; time: *F* (1, 38) = 6.213; *p* = 0.017; *η²* = 0.140; interaction: *F* (1, 38) = 1.873; *p* = 0.179; *η²* = 0.047).

First, a main effect test of the group factor revealed a significant difference at the pre-test (*F* (1, 38) = 13.546; *p* < 0.001) and post-test (*F* (1, 38) = 6.610; *p* = 0.014). Multiple comparisons using Bonferroni's method indicated that the mean scores for ON-PT were significantly higher than those for F2F-PT at the pre- and post-tests.

Second, a main effect test of the time factor (pre- and post-tests) at each level of the provision condition factor (F2F-PT and ON-PT) showed no significant difference for F2F-PT (*F* (1, 12) = 0.389; *p* = 0.544), but a significant difference for ON-PT (*F* (1, 26) = 12.642; *p* = 0.001). Multiple comparisons using Bonferroni's method revealed that the mean scores were significantly higher at the pre-test than at the post-test.

Child Domain of PSI

The results of the analysis for the child domain of PSI are presented in Table 3. The two-way RM-ANOVA revealed a significant main effect of group factor and interaction effect and a marginally significant main effect of time (group: *F* (1, 38) = 12.425; *p* = 0.001; *η²* = 0.246; time: *F* (1, 38) = 3.966; *p* = 0.053; *η²* = 0.094; interaction: *F* (1, 38) = 4.111; *p* = 0.049; *η²* = 0.097).

**Figure 2:**
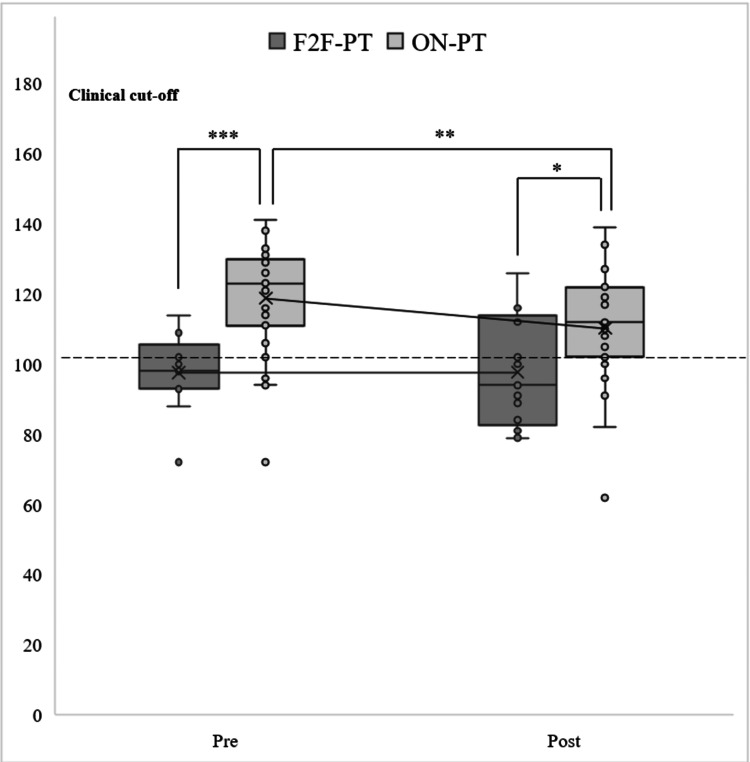
Multiple comparisons using Bonferroni's method (child domain of PSI). ^***^p < 0.001;^ **^p < 0.01; ^*^p < 0.05. F2F-PT: face-to-face parent training; ON-PT: online parent training; PSI: Parenting Stress Index

First, a simple main effect test of the group factor revealed a significant difference at the pre-test (*F* (1, 38) = 18.100; *p* < 0.001) and post-test (*F* (1, 38) = 5.244; *p* = 0.027). Multiple comparisons using Bonferroni's method indicated that the mean scores for ON-PT were significantly higher than those for F2F-PT at the pre- and post-tests (Figure 2).

Second, a simple main effect test of the time factor (pre- and post-tests) at each level of the provision condition factor (F2F-PT and ON-PT) showed no significant difference for F2F-PT (*F* (1, 12) = 0.000; *p* = 0.982), but a significant difference for ON-PT (*F* (1, 26) = 11.914; *p* = 0.001). Multiple comparisons using Bonferroni's method revealed that the mean scores were significantly higher at the pre-test than at the post-test (Figure 2).

Percentage of Cut-Off (PSI)

The proportions of participants meeting the cut-off scores are presented in Table 4. For the total score, the number of participants in the F2F-PT showed no change, with four (30.76%) at both pre- and post-tests, and in the ON-PT, the number decreased from 22 (81.48%) at the pre-test to 19 (70.37%) at the post-test.

For the parent domain of PSI, the number of participants in the F2F-PT increased from two (15.38%) at the pre-test to three (23.07%) at the post-test, and in the ON-PT, the number increased from 15 (55.55%) at the pre-test to 19 (70.37%) at the post-test. For the child domain of PSI, the number of participants in the F2F-PT increased from four (30.76%) at the pre-test to five (38.46%) at the post-test, and in the ON-PT, the number decreased from 25 (92.59%) at the pre-test to 24 (88.88%) at the post-test.

Children's behavioral problems

Total Score of ECBI

The results of the analysis for the total scores are presented in Table 3. The two-way RM-ANOVA revealed a significant interaction effect, a marginally significant main effect of group, and a non-significant main effect of time (group: *F* (1, 38) = 3.631; *p* = 0.064; *η²* = 0.087; time: *F* (1, 38) = 2.796; *p* = 0.102; *η²* = 0.068; interaction: *F* (1, 38) = 6.704; *p* = 0.013; *η²* = 0.150).

**Figure 3:**
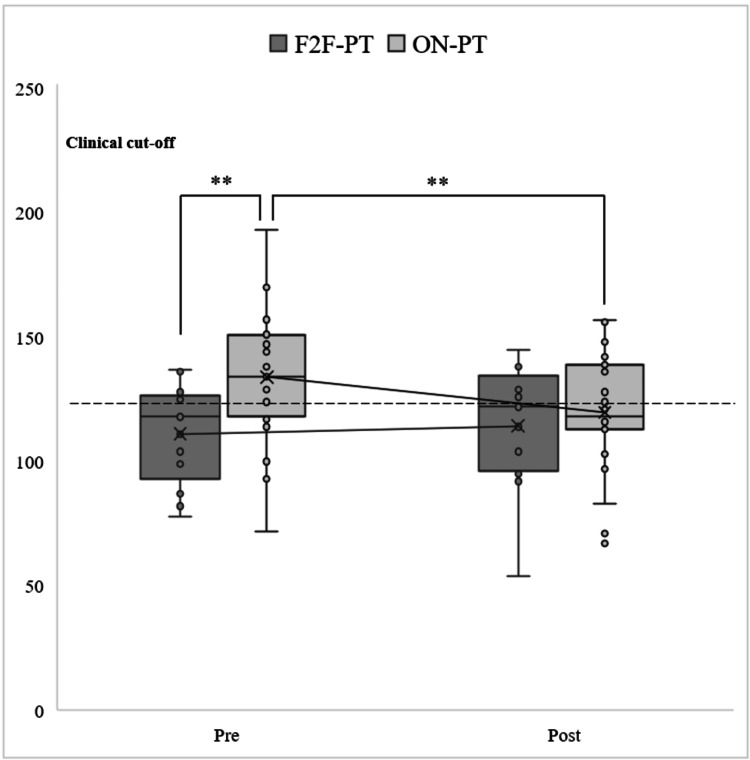
Multiple comparisons using Bonferroni's method (ECBI). ^**^p < 0.01. F2F-PT: face-to-face parent training; ON-PT: online parent training; ECBI: Eyberg Child Behavior Inventory

First, a simple main effect test of the group factor revealed a significant difference at the pre-test (*F* (1, 38) = 7.656; *p* = 0.008). Multiple comparisons using Bonferroni's method indicated that the mean scores for ON-PT were significantly higher than those for F2F-PT at the pre-test (Figure 3).

Second, a simple main effect test of the time factor (pre- and post-tests) at each level of the provision condition factor (F2F-PT and ON-PT) showed no significant difference for F2F-PT (*F* (1, 12) = 0.521; *p* = 0.484), but a significant difference for ON-PT (*F* (1, 26) = 11.780; *p* = 0.002). Multiple comparisons using Bonferroni's method revealed that the mean scores were significantly higher at the pre-test than at the post-test (Figure 3).

Percentage of Cut-Off (ECBI)

The proportions of participants meeting the cut-off scores are presented in Table 4. For the total score, the number of participants in the F2F-PT increased from four (30.76%) at the pre-test to six (46.15%) at the post-test, and in the ON-PT, the number decreased from 19 (70.37%) at the pre-test to 12 (44.44%) at the post-test.

## Discussion

This study retrospectively evaluated the effectiveness of the real-time type of ON-PT delivered via a web conferencing system in enhancing the parental mental health and reducing behavioral problems in children with DD compared to F2F-PT. The results demonstrated that ON-PT reduced parental stress and improved children's behavioral problems compared to F2F-PT.

The attendance rates for F2F-PT (82%) and ON-PT (80.3%) were similar, as were the dropout rates for F2F-PT (18.7%) and ON-PT (18.1%). The real-time type of ON-PT has the potential to maintain participant motivation by enhancing interaction with participants [18], and the findings of this study support this concept. In contrast, a large-scale randomized controlled trial (RCT) of an on-demand type of ON-PT reported that 31% of participants did not complete the program [15]. Therefore, the real-time type of ON-PT format may help overcome the challenges of maintaining participant motivation observed in the on-demand type [15].

Parental mental health showed a marginal improvement in PSI only in the ON-PT, with a significant improvement in the child domain of PSI. BDI-II scores showed a marginal improvement in the F2F-PT and a significant improvement in the ON-PT. The results suggest that ON-PT may be as effective as or more effective than F2F-PT in improving parental mental health. In contrast, studies on the on-demand type of ON-PT have not shown improvements in parental mental health [12]. The improvement in parental mental health observed in the real-time type of ON-PT in this study likely resulted from peer support [18] generated through participant communication, a benefit previously reported in F2F-PT [28]. Therefore, the real-time type of ON-PT may include elements that enhance parental mental health, which are absent in the on-demand type of ON-PT. Future research should qualitatively explore mechanisms through which peer support and the real-time type ON-PT enhance effectiveness.

Significant reductions in children's behavioral problems, as measured by the ECBI, were observed exclusively in the ON-PT. Furthermore, despite a higher proportion of participants being in the clinical range at pre-test in the ON-PT compared to the F2F-PT, the proportion decreased at post-test. The results of this study are consistent with findings from studies that directly compared the on-demand type of ON-PT to F2F-PT [12]. Therefore, ON-PT is considered potentially effective regardless of the delivery format (on-demand or real-time type), even when children's behavioral problems are in the clinical range at the start of the intervention. Future research should evaluate the effectiveness of ON-PT across diverse clinical populations and investigate its long-term effects.

This study provides preliminary evidence that ON-PT may be as effective as or more effective than F2F-PT. ON-PT is also widely supported, with 28.5% of Japanese parents in a PT survey identifying it as necessary [29]. Furthermore, ON-PT has the potential to substantially improve access to PT for families in remote areas, such as mountainous regions and islands, where traditional programs are inaccessible; further research is needed to evaluate its effectiveness. However, these findings should be interpreted cautiously due to several limitations of this study.

First, the retrospective study design lacked randomization, introducing potential selection bias and limiting the generalizability of results. Second, ON-PT was delivered via web conferencing under COVID-19 restrictions, which may have influenced intervention delivery and participant engagement compared to standard conditions. Third, differences in study periods for F2F-PT and ON-PT may have affected outcomes due to temporal variations. Fourth, both PT programs were largely similar, but the inclusion of different relaxation techniques may have influenced the results. Fifth, baseline differences in indicator scores between ON-PT and F2F-PT suggest potential imbalances in participant characteristics, complicating direct comparison. Notably, this study does not imply that F2F-PT is inferior to ON-PT; F2F-PT had a lower proportion of participants with high-risk levels of children's behavioral problems and parental mental health issues, whereas ON-PT had a significantly higher proportion, precluding strict comparisons. Future research should validate the effectiveness of ON-PT compared to F2F-PT using a prospective, rigorously controlled design. Lastly, the absence of data on PT acceptance in the F2F-PT precluded direct comparison with the ON-PT. A comprehensive assessment of PT effectiveness should include qualitative data to complement quantitative findings, as recommended by Gardner et al. [30].

## Conclusions

The study results suggest that ON-PT may be as effective as or more effective than F2F-PT. Given that the real-time type of ON-PT may reduce parental stress and improve children's behavioral problems compared with F2F-PT, therefore, adopting online formats should be actively considered for families facing these challenges. Moreover, since ON-PT can be delivered to families in remote areas, future programs should incorporate strategies that leverage its accessibility benefits. Nonetheless, the retrospective study design warrants caution in interpreting the findings. A future study with a prospective, rigorously controlled design and a mixed-methods approach is essential to effectively compare the effectiveness of ON-PT and F2F-PT. This design would enable the exploration of qualitative insights into parents' subjective experiences and preferences regarding PT modalities, providing a comprehensive understanding of factors influencing the effectiveness and acceptance of different PT formats.
